# Spatial variability in the diversity and structure of faunal assemblages associated with kelp holdfasts (*Laminaria hyperborea*) in the northeast Atlantic

**DOI:** 10.1371/journal.pone.0200411

**Published:** 2018-07-12

**Authors:** Harry Teagle, Pippa J. Moore, Helen Jenkins, Dan A. Smale

**Affiliations:** 1 Marine Biological Association of the United Kingdom, The Laboratory, Citadel Hill, Plymouth, United Kingdom; 2 Ocean and Earth Science, National Oceanography Centre Southampton, University of Southampton, Southampton, United Kingdom; 3 Institute of Biological, Environmental and Rural Sciences, Aberystwyth University, Aberystwyth, United Kingdom; 4 Centre for Marine Ecosystems Research, School of Natural Sciences, Edith Cowan University, Joondalup, Western Australia, Australia; University of Sydney, AUSTRALIA

## Abstract

Kelp species are ecologically-important habitat-formers in coastal marine ecosystems, where they alter environmental conditions and promote local biodiversity by providing complex biogenic habitat for an array of associated organisms. While it is widely accepted that kelps harbour significant biodiversity, our current understanding of spatiotemporal variability in kelp-associated assemblages and the key environmental drivers of variability patterns remains limited. Here we examined the influence of ocean temperature and wave exposure on the structure of faunal assemblages associated with the holdfasts of *Laminaria hyperborea*, the dominant habitat-forming kelp in the northeast Atlantic. We sampled holdfasts from 12 kelp-dominated open-coast sites nested within four regions across the UK, spanning ~9° in latitude and ~2.7° C in mean sea surface temperature. Overall, holdfast assemblages were highly diverse, with 261 taxa representing 11 phyla recorded across the study. We examined patterns of spatial variability for sessile and mobile taxa separately, and documented high variability between regions, between sites within regions, and between replicate holdfasts for both assemblage types. Mobile assemblage structure was more strongly linked to temperature variability than sessile assemblage structure, which was principally structured by site-level variability in factors such as wave exposure. Patterns in the structure of both biogenic habitat and associated assemblages did not vary predictably along a latitudinal gradient in temperature, indicating that other processes acting across multiple spatial and temporal scales are important drivers of assemblage structure. Overall, kelp holdfasts in the UK supported high levels of diversity, that were similar to other kelp-dominated systems globally and comparable to those recorded for other vegetated marine habitats (i.e. seagrass beds), which are perhaps more widely recognised for their high biodiversity value.

## Introduction

In the marine environment, the distribution of species and the structure of communities are regulated by a range of biotic and abiotic factors that operate over multiple spatial and temporal scales [1–6]. Understanding the relative influence of key processes in structuring populations and communities is a central goal of ecology, and is of elevated importance given that abiotic and biotic factors are being altered by human activities [7–12]. By examining patterns of variability in ecological structure over multiple spatial and temporal scales, insights can be gained into the relative importance of processes that vary across similar scales. For example, repeated regional-scale observations conducted across latitudinal gradients in ocean temperature can elucidate the influence of temperature on the ecophysiological performance of populations and species [13, 14], the biogeographical distributions of species [15–17] and, in turn, the structure of communities [14, 18–20]. Similarly, examining biodiversity patterns across smaller spatial scales that encapsulate natural gradients in other factors (e.g. wave exposure, grazing pressure, turbidity) can provide insights into their relative importance in structuring communities [21–27]. Establishing baselines of biodiversity patterns at multiple scales within key ecosystems is vital, given the rate at which species’ distributions and abundances are changing in the current period of anthropogenic environmental change [28, 29].

Foundation species exert strong influence over other organisms by altering environmental conditions and, in many cases, creating or modifying habitat for other species [30, 31]. Kelps, large brown seaweeds of the order Laminariales, are the dominant foundation species along temperate and subpolar rocky coastlines in both hemispheres [9]. Kelps are amongst the fastest growing autotrophs on Earth [32–34] and, as such, represent a major source of primary production and an important food source in coastal environments [35, 36]. Kelps also promote secondary productivity through the provision of three-dimensional habitat structure, which supports a myriad of associated organisms including species of commercial and ecological importance [8, 9, 37]. They support increased levels of biodiversity by offering greater habitat space, heterogeneity and complexity, as well as through direct and indirect (via epibionts) provision of food [38]. A recent review of the role of kelps as biogenic habitat formers found that individual plants often support highly abundant invertebrate assemblages, often numbering in the high tens of thousands per kelp plant (see [38] and references therein). As habitat formers, mature kelp thalli provide three distinct micro-habitats; the blade (lamina), the stipe, and the holdfast, all of which differ considerably in form and structure, and consequently support assemblages of differing composition, diversity and abundance [38]. Of these micro-habitats, the holdfast generally supports the most diverse and temporally persistent assemblage, and has received greatest attention in the literature (see [38] and references therein).

The interstitial space between the underlying hard substratum and the haptera (the root-like structures which form the holdfast) of kelp holdfasts represents favourable habitat for colonising fauna, primarily because (i) the surface area and volume of habitat available for colonisation is increased; (ii) the structure offers protection from adverse environmental conditions and predators, and (iii) food availability is enhanced through accumulation of organic matter [39–42]. In general, the living space within kelp holdfasts offers a range of niches that may differ from adjacent habitats. Assemblages associated with kelp holdfasts are often diverse and abundant, with up to 90 macrofaunal species [2, 43, 44] and 10,000 individuals inhabiting a single holdfast [2, 45]. Variability in the structure of holdfast-associated assemblages is driven by a range of biotic and abiotic factors, including the size and complexity of the holdfast itself [2, 46–48], hydrodynamic forces [49, 50], sedimentation rates and sediment content [45, 51], food and larval supply [25, 52], pollution [53–55], turbidity [25, 56] and depth [51, 57]. In addition, kelps are cool-water species and the structure of kelp populations is known to vary along large-scale gradients in ocean temperature [13, 58]. Marginal equatorward populations, in particular, are stressed by increases in temperature [59, 60] and, as such, observed and predicted ocean warming trends will likely impact upon kelp populations and affect their functioning as habitat-forming foundation species. It seems likely, therefore, that ocean climate will be a key driver of holdfast assemblage structure, because (i) biogenic habitat structure is likely to vary with temperature as kelp populations respond to climatic conditions, (ii) the biogeographic distributions of marine species are strongly constrained by temperature [17] and (iii) the structure of populations of kelp-associated fauna (i.e. abundances) is likely to vary with ocean climate [61]. However, as very few studies have examined variability in holdfast assemblages across spatial scales large enough to encompass natural temperature gradients, the influence of ocean climate on biodiversity patterns remains unclear.

**Fig 1:**
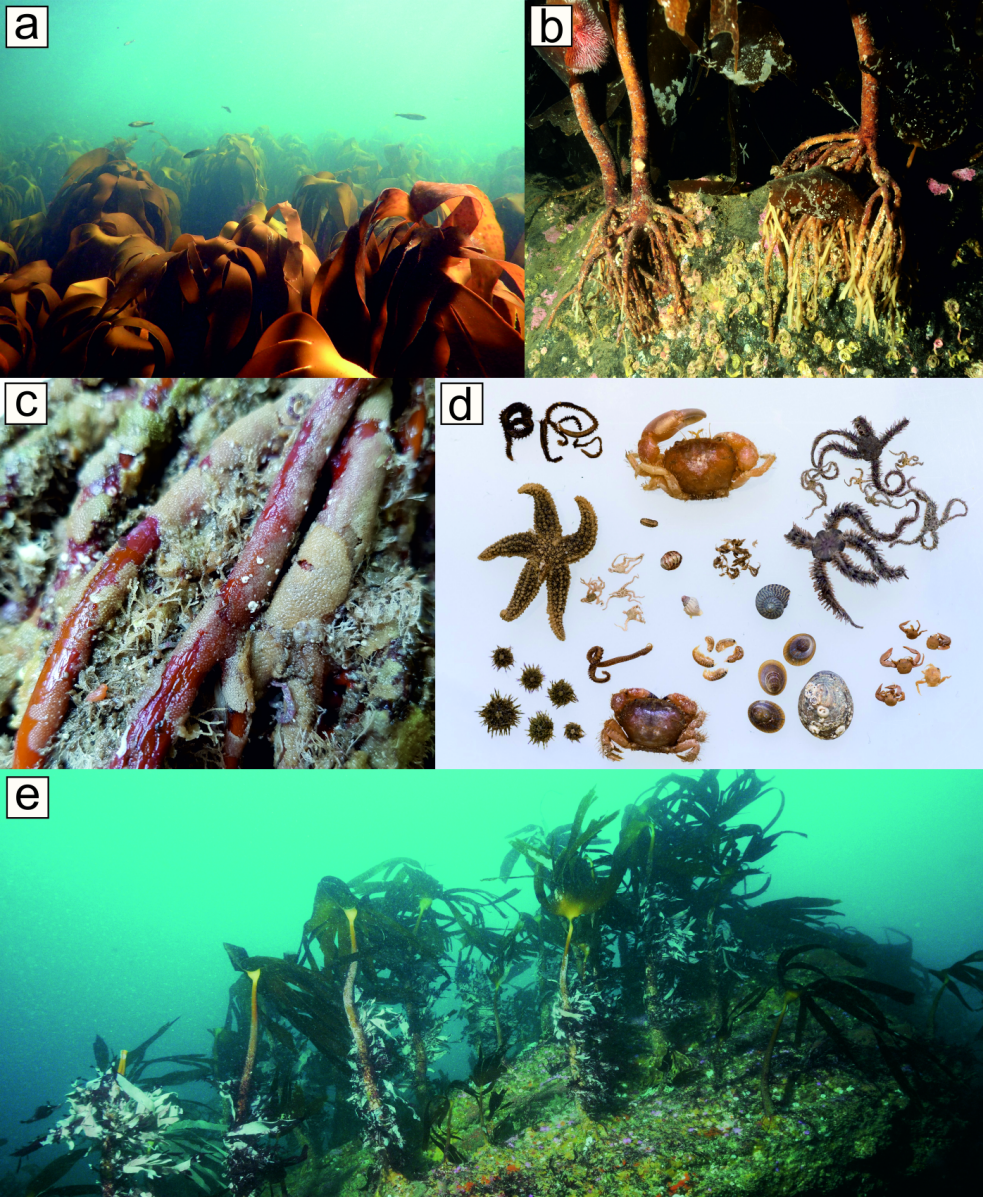
(a) *Laminaria hyperborea* is a dominant kelp species in the northeast Atlantic, where it forms dense, extensive macroalgal canopies. (b) It forms a large, complex holdfast structure, which anchors the plant to rocky substrata and provides biogenic habitat for associated organisms. (c) Holdfasts sampled were typically encrusted by a high coverage of sessile invertebrates. (d) The interstitial space between the reef surface and the holdfast was utilised by a high diversity of mobile invertebrates. (e) Dense stands of *L*. *hyperborea* may serve as ecologically-significant repositories of biodiversity.

In the northeast Atlantic, wave-exposed subtidal rocky reefs are generally dominated by the kelp *Laminaria hyperborea* (Gunnerus) Foslie 1884; a large, stipitate kelp which attaches to rocky substratum by a well formed, typically ‘laminarian’ holdfast [38], from the extreme low intertidal to depths of up to 40 m in clear oceanic waters [62]. *L*. *hyperborea* is a boreal species, distributed from northern Portugal to its poleward range edge in northern Norway, Iceland and the Russian Murmansk coast. *L*. *hyperborea* is the foremost canopy former on shallow, wave exposed rocky reefs throughout this region [63, 64], and represents an important habitat for coastal biodiversity and other ecosystem services [8]. In the UK, *L*. *hyperborea* is spatially extensive, forms dense canopies and offers a high quantity and quality of biogenic habitat (Fig 1). In general, however, kelp ecosystems in the UK have been relatively understudied since the pioneering work of the 1960s and 70s (e.g. [34, 44, 65]), particularly when compared to the volume of work conducted in other research-intensive nations (e.g. Australia and the USA; [8]). Perhaps surprisingly, fundamental information on biodiversity patterns associated with *L*. *hyperborea*, and the potential multi-scale drivers of variability in holdfast assemblage structure, are still lacking.

Here, we examined the structure of holdfast assemblages at 12 sites within 4 regions of the UK to better understand spatial variability in kelp-associated biodiversity. The specific aims of the study were to (1) benchmark the diversity, abundance, biomass, and structure of holdfast assemblages across the latitudinal distribution of *L*. *hyperborea* in the UK; (2) examine multiscale spatial variability patterns in holdfast assemblage structure; and (3) explicitly link variability in ecological pattern with potential environmental drivers. Recent work on the kelp canopies themselves has documented high between-site variability in population and habitat structure, as well as regional differences between northerly and southerly locations [19, 58]. Given that holdfast assemblages are likely to be strongly influenced by the population structure of host kelp species [38, 46, 66], we predicted that they would also exhibit high levels of small-scale variability (i.e. between plants and sites), as well as some structuring across larger spatial scales (i.e. between regions).

## Methods

### Study area

**Fig 2:**
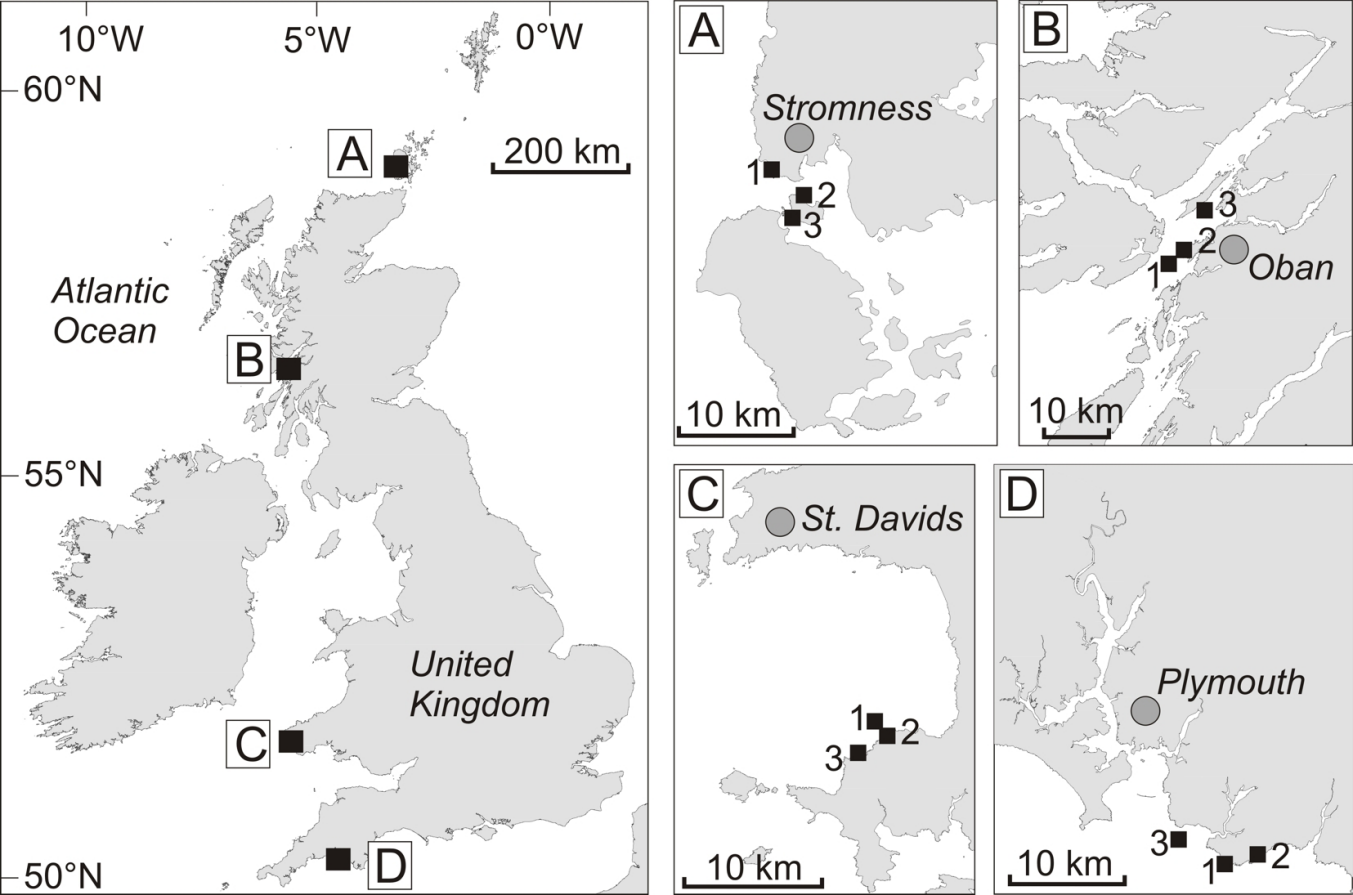
Map indicating the locations of the four study regions in the UK, northeast Atlantic: (A) northern Scotland, (B) western Scotland, (C) southwest Wales and (D) southwest England. Smaller panels show the positions of the 3 study sites within each region.

*Laminaria hyperborea* holdfasts were sampled by scuba divers from 12 sites nested within four regions in the UK (Fig 2); north Scotland (region ‘A’), west Scotland (B), southwest Wales (C) and southwest England (D). All sites were located on the exposed west coast of the UK, where kelp forest habitat is abundant, and span 9° of latitude (~50° to ~59° N), and encompass a temperature gradient of ~2.7°C (Table 1). All study sites within these regions were ‘open coast’, moderately to fully exposed to wave action and were characterised by extensive subtidal rocky reef at depths of 0 to >5 m (below chart datum). All sites were also deemed to be representative of the wider region, in terms of coastal geomorphology, and were not influenced by local anthropogenic activities.

**Table 1:** Predictor variables recorded at 12 study sites within 4 distinct regions in the UK. ‘Mean SST’ is the annual mean temperature calculated from satellite-derived sea surface temperature (SST) data (2005–2014). ‘Log wave fetch’ is a broad-scale metric of wave exposure, derived by summing fetch values calculated for 32 angular sectors surrounding each site (see [71]). ‘Log chl *a* mean’ is the average annual concentration of chlorophyll *a* (log10 mg m^-3^ from MODIS Aqua satellite data, 2002–2012). ‘Peak summer max (mean) temp’ is the maximum (average) daily temperature recorded between 26 July and 18 August 2014, when all sensor array deployments overlapped. ‘Summer daylight’ is the average daytime (08:00–20:00) light intensity during a 14 d deployment of light loggers. ‘Tidal water motion’ is a proxy for water movement driven by tidal flow, derived from the range in water motion values recorded during a 24 h period, averaged over the 45 d accelerometer deployment. ‘Wave water motion’ is a proxy for water movement driven by waves, derived from averaging the 3 highest-magnitude water motion values observed during the 45 d accelerometer deployment (following correction for tidal-movement). ‘PO_4_^3-^‘ and ‘NO_3_^-^ +NO_2_^-^‘ indicate averaged spring and summer concentrations of phosphate and nitrite + nitrate respectively (n = 4 water samples taken from ~1 m above the kelp canopy).

Region	Site	Locality	Mean SST (°C)	Log wave fetch (km)	Log chl *a* mean (mg m^-3^)	Peak summer temp (°C)		Summer daylight (lumens m^-2^)	Tidal water motion (m s^-1^)	Wave water motion (m s^-1^)	NO_3_^-^+NO_2_^-^ (μM)	PO_4_^3-^ (μM)
						Max	Mean					
**N Scot (A)**	A1	Warbeth	9.7	3.8	0.21	13.99	13.69	7124	0.18	1.02	1.66	0.17
	A2	N Graemsay	9.8	3.5	0.26	13.68	13.49	4835	0.20	0.30	1.92	0.23
	A3	S Graemsay	9.7	3.4	0.26	13.87	13.65	5144	0.26	0.16	2.58	0.19
**W Scot (B)**	B1	Dubh Sgeir	10.8	3.3	0.59	13.96	13.69	4794	0.15	0.22	2.93	0.30
	B2	W Kerrera	10.7	3.1	0.65	13.93	13.68	3094	0.05	0.08	2.52	0.26
	B3	Pladda Is.	10.8	2.8	0.73	14.52	14.06	4874	0.19	0.11	1.96	0.27
**Wales (C)**	C1	Stack Rock	11.7	3.7	0.43	17.06	16.54	1861	0.13	0.73	3.17	0.16
	C2	Mill Haven	11.8	3.5	0.43	17.15	16.62	3657	0.08	0.34	2.68	0.22
	C3	St. Brides	11.8	3.4	0.43	17.13	16.63	2960	0.08	0.23	3.09	0.14
**SW Eng (D)**	D1	Hillsea Pt.	12.4	4.1	0.28	17.62	16.80	2746	0.15	0.42	3.41	0.17
	D2	E Stoke Pt.	12.4	3.9	0.28	18.31	17.09	2840	0.11	0.22	2.41	0.11
	D3	N Mewstone	12.5	3.5	0.38	17.71	17.06	4432	0.06	0.20	3.05	0.35

### Environmental variables

Environmental sensors were deployed on a small sub-surface buoy, suspended in the water column immediately above the reef surface by a 0.65 m rope, which was attached to a clump weight and allowed free movement of the sensor in response to water motion. Sensors were deployed within a 4-week period in July and August 2014 and retrieved ~6 weeks later. Each sensor array was deployed for >45 days at each site, and any surrounding kelp plants (~2 m radius) were removed to negate any shading and/or impediment of water movement around the buoy. These arrays recorded temperature, light levels, and relative water motion at fine temporal resolutions. In order to quantify water motion driven by wave exposure and/or tidal regimes, an accelerometer (HOBO Pendant G Logger, Onset, attached to the buoy) recorded its position in three axes every 5 minutes (see [67]). A temperature and light level sensor was also attached to the buoy and captured data every 15 minutes (see [58] for further details). Relative water motion was calculated from the accelerometer data by extracting movement data in the planes of the x- and y-axes, after first subtracting the modal average of the entire dataset from each value to account for any latent static ‘acceleration’ resulting from the accelerometer not sitting exactly perpendicular to the seabed (caused by imprecise attachment of the logger to the buoy or the buoy to the tether).

Accelerometer data were converted to water motion following Evans and Abdo [68], and then used to generate two separate metrics, one for water movement induced by tidal flow, and one by wave action. For tidal flow, all values above the 90^th^ percentile (i.e. those most likely related to turbulent, wave driven water movement) were removed. The range of water motion values recorded within each 12 h period (representing ~1 complete tidal cycle) was then calculated and averaged over the 45 d deployment. Following subtraction of average water motion induced by tidal flow, wave-induced water movement was calculated by taking an average of the 3 highest magnitude values recorded for each site. Recorded temperature data was converted to daily mean temperatures; a 24 h period during peak summer temperatures, where all sensor array deployments overlapped was then used to generate maximum daily means and average daily temperature for each study site. To establish average summer daytime (08:00–20:00 h) light levels for each site, data for the first 14 d of deployment were used, in order to limit the potential of fouling by biofilms and epiphytes to affect light measurements.

In addition, nutrient levels at each site were assessed by collecting 2 independent seawater samples from immediately above the kelp canopy with duplicate 50 ml syringes. Samples were passed through a 0.2 μm syringe filter and kept on ice without light, before being frozen and analysed (within 2 months) for nutrients (nitrate+nitrite; NO_3_^-^+NO_2_^-^, and phosphate; PO_4_^3-^) using standard analytical techniques (see [69] and references therein). Nutrient concentrations were quantified twice at each site (in summer 2014 and spring 2015) and mean values are presented here. In addition to these fine-scale variables, average sea temperatures (2005–2014) were calculated from satellite-derived SSTs (9 km resolution AVHRR data), wave fetch was calculated for each site following Burrows et al. [70] and Burrows [71], and estimates of chlorophyll *a* (chl *a*) concentrations were generated from optical properties of seawater derived from satellite images (collected by the MODIS Aqua satellite at 9 km resolution, averaged for the period 2002 to 2012).

### Sample collection and processing

Six holdfast samples were collected in late summer (i.e. August/September 2014) from each site (72 samples in total). Mature, canopy forming *L*. *hyperborea* plants (see Fig 1) were haphazardly selected from within dense kelp stands at depths of 2–4 m (below chart datum). Divers cut the stipe of each plant just above the holdfast, which was immediately covered with a fine-mesh cotton bag to prevent the loss of mobile fauna, and then gently prised the holdfast from the reef before sealing the bag with a cable tie. Kelp plants were situated >2 m apart and samples were all individual, rather than fused, holdfasts. Samples were immediately treated with a 1% propylene phenoxytol solution for ~30 minutes, in order to relax soft invertebrate specimens to aid in later identification, and stored in 70% industrial methylated spirit (IMS) solution until processing. To process, holdfasts were rinsed with freshwater, and any mobile fauna was collected in a 1 mm sieve and returned to 70% IMS solution for subsequent identification. The total holdfast volume was then quantified by displacement; the entire holdfast structure was wrapped in plastic kitchen wrap and submerged in freshwater. Haptera (the root-like structures which make up the holdfast) were then removed to reveal the internal holdfast structure and any sessile fauna within, which were identified immediately. The volume of the cleaned haptera were then measured (using displacement), subsequently being subtracted from total holdfast volume to give the volume of the potential habitable space within the holdfast, and wet weight was recorded. All fauna was identified to the lowest taxonomic level possible, in most cases species (~67% of taxa). Sessile fauna was weighed (tissue-dried fresh weight) to establish biomass, whereas mobile fauna were enumerated for abundance. Finally, each kelp sampled was aged by sectioning the stipe immediately above the holdfast and counting seasonal growth rings, as described by Kain [65]. All sampling was conducted according to UK legislation and under the guidance of Natural England, Scottish Natural Heritage and the Inshore Fisheries and Conservation Authorities. No specific permits were required to collect samples as all study sites fall outside protected or legislated areas, and no protected species were collected.

### Statistical analysis

All analysis was conducted using univariate and multivariate permutational analyses using the PERMANOVA add on [72] for Primer v7 software [73]. Metric multidimensional scaling (mMDS) ordinations were constructed to visualise multivariate patterns. Variability in assemblage structure was examined with multivariate PERMANOVA using a 2-factor design, with region (4 levels) as a fixed factor and site (3 levels) as a random nested factor. To examine correlations with habitat size, habitable holdfast space was included as a co-variate in all analyses. Permutations (4999 under a reduced model) were based on a Bray-Curtis similarity matrix constructed from fourth root transformed biomass data (for sessile assemblages) and fourth root transformed abundance data (for mobile assemblages). Fourth root transformation was chosen to down weight the influence of large sponges and colonial ascidians and high abundances of amphipods, respectively. Pair-wise tests between regions were conducted wherever the main effect was significant (P <0.05). Differences in multivariate dispersion between assemblages were examined using the PERMDISP routine. Where significant differences in assemblage structure between regions were detected, SIMPER analysis was performed to determine which taxa contributed most to the observed dissimilarity. Assemblages were first standardised by habitable holdfast space to account for variability related to habitat size. Univariate assemblage metrics (i.e. total biomass and species richness) were examined using the same model, but with permutations based on resemblance matrices generated from Euclidean distances between untransformed data. The biogenic structure of holdfasts (i.e. total volume, living space, age) was examined using the same univariate model but without the co-variate. PERMDISP was again used to examine differences in within-group (univariate) dispersion between levels of the factor of interest.

Relationships between environmental variables and assemblage structure were examined using the DISTLM (distance-based linear models) routine in PERMANOVA. Predictor variables included the environmental data shown in Table 1, as well as the habitat characteristics of holdfast age and habitable space. Prior to analysis, draftsman’s plots were generated from normalised data, and Pearson’s correlation coefficient was used to check for colinearity between variables. All temperature measures were highly correlated (r > 0.9), so only mean summer temperature was retained in the analysis. Mean nitrate+nitrite (NO_3_^-^+NO_2_^-^) and chl *a* concentration were also highly correlated with other variables and were excluded from the analysis. The DISTLM routine was then used to obtain the most parsimonious model using a stepwise selection procedure and AICc selection criterion [72, 74].

To examine the biogeographic affinities of individual species comprising holdfast assemblages, all fauna identified to species level (174 species) were classified based on the southernmost limit (i.e. warm-water equatorward range edge) of their recorded distributions. Species were categorised in bins of 10° latitude (which ranged from the ‘warmest’ bin of 20°-30°N to the ‘coolest’ bin of 50°-60°N) based on data from the Ocean Biogeographic Information System (OBIS), the Global Biodiversity Information Facility (GBIF), and the World Register of Marine Species (WoRMS). We hypothesised that our northernmost study regions would support a greater proportion of species with higher latitude equatorward range edges (i.e. Arctic or Boreal species) whereas southernmost regions would support more species with lower latitude equatorward range edges (i.e. Lusitanian species).

Finally, we explicitly compared spatial variability patterns between sessile and mobile assemblages. We used the RELATE procedure in Primer v7 to measure correlations between similarity matrices generated from sessile and mobile assemblages at different spatial scales (i.e. the full datasets at the holdfast level, as well as datasets averaged to the site level and to the region level). The technique uses Spearman Rank correlation to determine the relatedness of two similarity matrices, with a ρ value of 1 indicating that dissimilarity patterns are entirely correlated. We also examined correlations in taxon richness and total biomass/abundance between sessile and mobile assemblages using scatterplots and Pearson’s correlation tests.

## Results

### Environmental variables

Seawater temperature differed across study regions, with a clear distinction between northern (A, B) and southern regions (C, D; Table 1). Mean sea temperatures in southern regions were ~2.7°C higher than in northern regions. Wave fetch was generally comparable between sites and regions, although the greatest values were recorded for sites in northern Scotland and southwest England (Table 1). Water motion associated with tidal flow was most pronounced in northern Scotland (sites A2 and A3). Light intensity was variable, both within and among regions (Table 1), with maximum light intensity (site A1) ~3 times greater than minimum light intensity (site C1). Nitrate and nitrite (NO_3_^-^+NO_2_^-^) and phosphate (PO_4_^3-^) concentrations were broadly comparable across sites and regions, although nitrate and nitrite values were slightly higher in Wales and southwest England and phosphate values were higher in west Scotland (Table 1).

### Biogenic habitat structure

**Fig 3:**
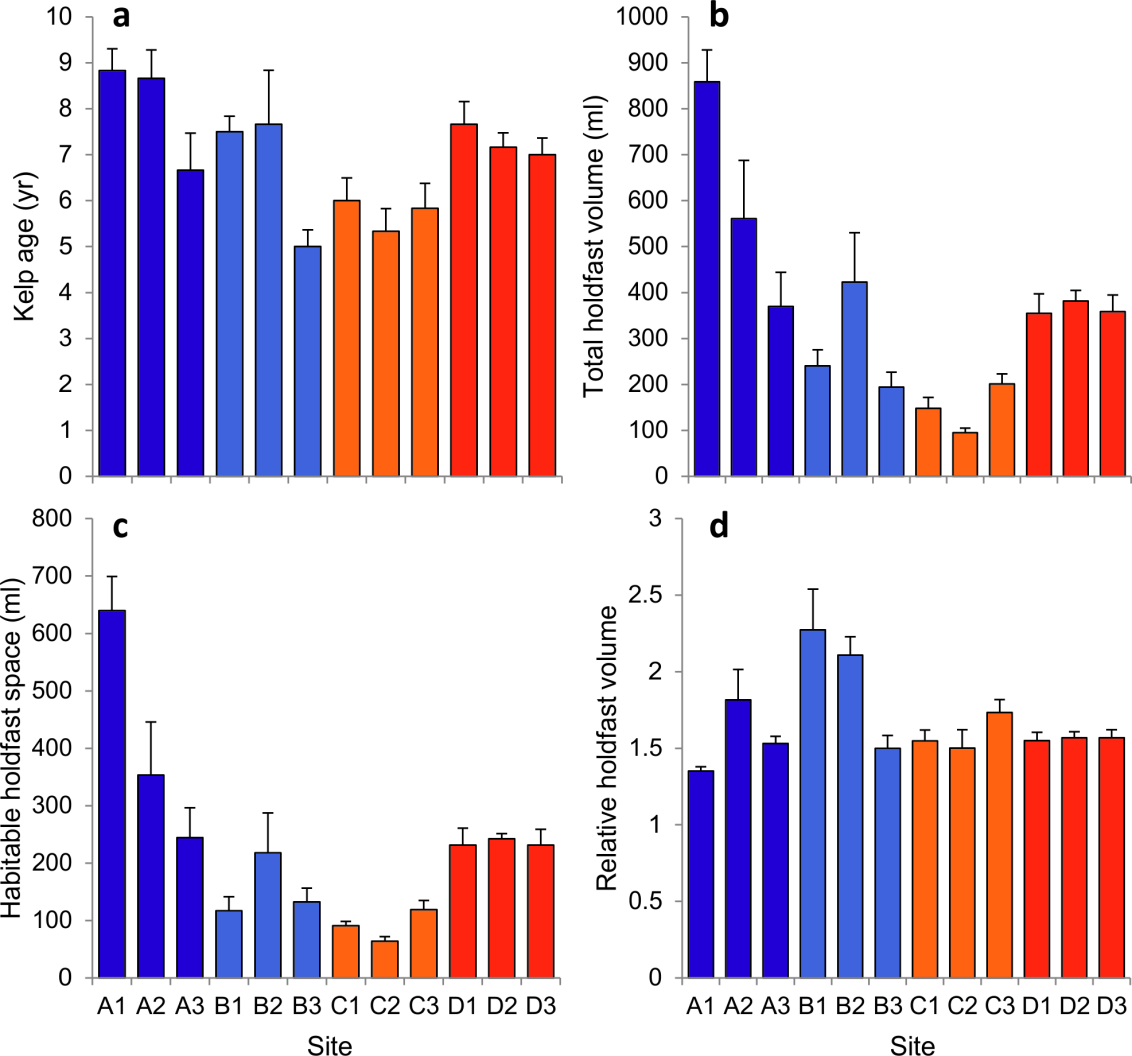
Biogenic habitat structure provided by *Laminaria hyperborea* holdfasts: (a) kelp age, (b) total holdfast volume (THV), (c) habitable holdfast space (HHS) and (d) relative holdfast space (THV/HHS). Values are means of 6 replicate holdfasts per site (± SE).

The average age of kelp plants varied to some degree (Fig 3A), with a maximum mean age of 8.83 ± 0.48 SE recorded in north Scotland (site A1) and a minimum mean age of 5 ± 0.37 SE in west Scotland (site B3). Overall, the age of individual kelp plants ranged from 4 years (sites C2, B2 and B3) to 12 years (site B2). Statistically, significant differences between sites were detected, but there was no overall effect of region (Table 2). Regional-scale variability was more pronounced for both total volume and habitable space (i.e. the living space contained within the holdfast), which tended to be highest in north Scotland and lowest in southwest Wales (Fig 3B and 3C). Significant variability was recorded both between regions and between sites (Table 2). Pairwise tests within the significant region factor showed that holdfast size (in terms of both total holdfast volume and habitable space) differed significantly between all regions, with the exception total holdfast volume at west Scotland and southwest England, where no difference was observed. In general, holdfasts from north Scotland (sites A1 and A2) were the largest observed, with those from southwest Wales being consistently the smallest (Fig 3B and 3C). The ratio of total volume to habitable space provided an estimate of internal complexity, with a higher ratio indicating relatively greater interstitial spacing. This metric was more consistent across regions, but did exhibit marked between-site variability in north and west Scotland (Fig 3D). Statistically, variability between sites was significant but no effect of region was detected (Table 2).

**Table 2:** Results of univariate PERMANOVA to test for differences in habitat metrics (a. kelp age, b. total holdfast volume, c. habitable holdfast space and d. relative holdfast space). Permutations were based on a Euclidean distance similarity matrix generated from untransformed data. All tests used a maximum of 4999 permutations under a reduced model; significant effects (P<0.05) are shown in bold. An underlined P-value indicates that PERMDISP detected significant differences in within-group dispersion between levels of that factor (P<0.05).

Source	*df*	MS	F	P	*df*	MS	F	P
	**a. Kelp age**				**b. Total holdfast volume**			
Region	3	17.333	2.943	0.123	3	6.36E+05	5.404	**0.007**
Site(Region)	8	5.8889	2.928	**0.007**	8	1.18E+05	5.216	**0.001**
Residual	60	2.0111			60	22551		
Total	71				71			
	**c. Habitable holdfast space**				**d. Relative holdfast space**			
Region	3	3.48E+05	5.105	**0.002**	3	0.6768	1.901	0.212
Site(Region)	8	68098	5.864	**0.001**	8	0.3544	4.412	**0.001**
Residual	60	11612			60	0.0803		
Total	71				71			

### Overall biodiversity patterns

Across the study (72 holdfasts), 261 taxa representing 11 phyla were recorded, with just over 70% classed as mobile fauna (S1 Table). In total 146 taxa were recorded from north Scotland (Region A), 134 taxa from western Scotland (B), 142 taxa from southwest Wales (C) and 136 from southwest England (D). Overall, taxa exhibited limited regional-scale specificity, with 33.7% of taxa being recorded in more than one region and 20.7% recorded in all four study regions. Over 8,000 individual mobile organisms were identified and counted, and the sessile fauna identified weighed ~500g.

### Sessile assemblages

**Fig 4:**
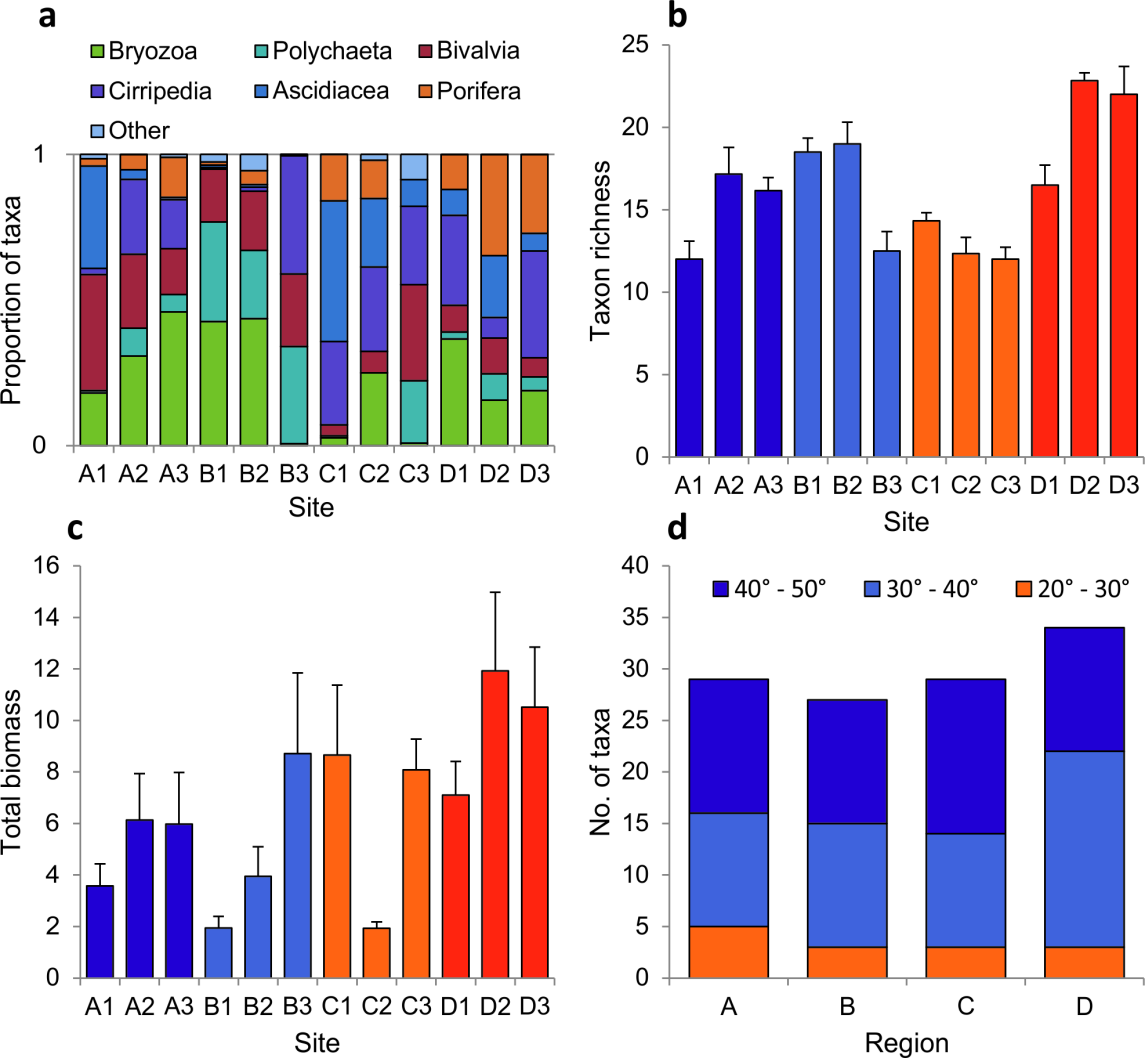
Univariate assemblage-level metrics for sessile holdfast assemblages: (a) the proportion of major taxonomic groups, (b) sessile assemblage taxon richness, (c) total biomass of sessile organisms, (d) taxa equatorward range edge. Values for (b) and (c) are means of 6 replicate holdfasts per site (±SE).

In total, 74 taxa were recorded from the 72 holdfasts (S1 Table). Typically, the haptera structures forming the holdfast were colonised by a high coverage of sessile organisms (Fig 1). Taxon richness varied from nine species per holdfast (samples from sites B3 and A1) to 25 species per holdfast (samples from site D3). The sessile assemblage was dominated by bryozoans (35 taxa), with the remainder comprised of bivalve molluscs (11 taxa), hydroids (eight taxa), barnacles (six taxa) and polychaetes (three taxa). Porifera (nine groups) were identified to morphological groups, while ascidians (split into colonial and solitary) and anthozoa (anemones) were broadly grouped, due to difficulty in identifying these taxa to species level. The proportion of major taxonomic groups found in the holdfasts was more or less consistent across sites, with the exception of sites B3 and C3, which lacked the high biomass of bryozoans characteristic of other sites, and of site C1, where holdfasts supported a high biomass of ascidians, and very low biomass of polychaetes and bryozoans (Fig 4A).

**Fig 5:**
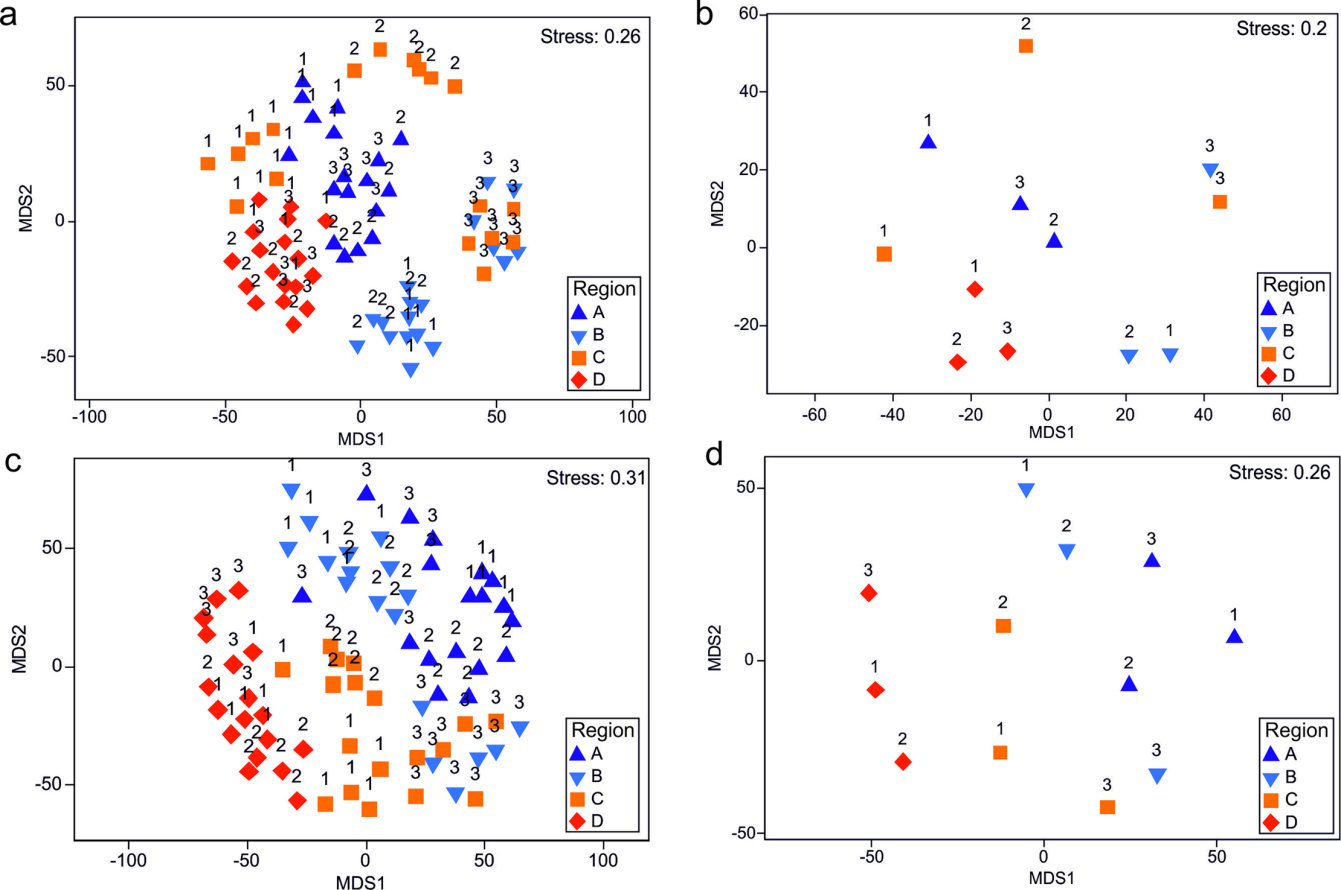
mMDS plots depicting the structure of sessile faunal assemblages, with centroids representing (a) individual holdfast samples (b) and site averages. Similarly mMDS plots depicting the structure of mobile faunal assemblages, with centroids representing (c) individual holdfasts and (d) site averages). Labels indicate sites and symbols indicate regions.

Metric MDS plots indicated some degree of partitioning between regions and, in some cases, between sites within regions (Fig 5A). Sites within regions A and D were distinctly grouped, but regions B, and particularly C, exhibited considerable variability between sites (Fig 5A). There was limited evidence of partitioning in structure between the cool northern regions and the warmer southern regions, even when centroids were averaged by site (Fig 5B). PERMANOVA detected significant variability between regions and between sites nested within regions, as well as a significant effect of the co-variate (Table 3). Pairwise tests within the region factor showed that assemblages in north Scotland were distinct from those in west Scotland and southwest England, which were also dissimilar from one another. PERMDISP showed significant differences between regions in within-factor multivariate dispersion (F_3,86_ = 29.22, P < 0.001), with variability between holdfast samples from Wales (region C) considerably greater than elsewhere.

**Table 3:** Results of multivariate PERMANOVA to test for differences in holdfast sessile (a) and mobile (b) assemblage between regions (fixed) and sites (random, nested within region). Habitable holdfast space (HHS) was included as a covariable in the analysis. Permutations were based on a Bray-Curtis similarity matrix generated from fourth-root transformed biomass/abundance data. Results of univariate PERMANOVA to test for differences in assemblage-level univariate metrics (taxon richness and total biomass) in holdfast assemblages are also shown (c–f). Permutations for univariate analysis were based on a Euclidean distance similarity matrix generated from untransformed diversity data. All tests used a maximum of 4999 permutations under a reduced model; significant effects (P<0.05) are shown in bold. An underlined P-value indicates that PERMDISP detected significant differences in within-group dispersion between levels of that factor (P<0.05).

Source	a. Sessile Assemblage				b. Mobile Assemblage			
	*df*	MS	*F*	P	*df*	MS	*F*	P
HHS	1	9222.1	2.084	**0.027**	1	9555.6	1.822	**0.017**
Region	3	10942	1.935	**0.031**	3	14436	2.219	**0.005**
Site(Region)	8	5824.1	10.83	**0.001**	8	6682.7	5.329	**0.001**
Residual	59	537.67			59	1254.1		
Total	71				71			
	**c. Sessile Taxon Richness**				**d. Mobile Taxon Richness**			
HHS	1	24.958	0.534	0.477	1	49925	5.705	**0.032**
Region	3	176.35	2.954	0.116	3	1733.9	0.178	0.867
Site(Region)	8	61.503	10.48	**0.001**	8	9942.1	650.1	**0.001**
Residual	59	5.8712			59	15.294		
Total	71				71			
	**e. Sessile Total Biomass**				**f. Mobile Total Abundance**			
HHS	1	91.856	4.230	0.062	1	3.09E+05	10.74	**0.005**
Region	3	99.772	3.581	0.065	3	43348	1.389	0.310
Site(Region)	8	28.714	12.67	**0.001**	8	31763	4.681	**0.001**
Residual	59	2.2670			59	6785.5		
Total	71				71			

SIMPER analysis indicated that the observed differences between regions were driven primarily by a higher biomass of sponges (Demosponge A and F), colonial ascidians (Didemnidae) and the barnacle *Verruca stroemia* in southwest England compared to west and north Scotland (S2 and S3 Tables). Holdfasts in west Scotland typically supported a high biomass of the barnacle *Balanus crenatus* but lacked the bryozoan *Celleporina caliciformis*, which was common in both north Scotland and southwest England (S2 and S3 Tables).

Taxon richness varied between sites within regions but, in general, the values tended to be highest in southwest England and lowest in Wales (Fig 4B). Total biomass exhibited high variability between replicate holdfasts and between sites, but no clear regional patterns (Fig 4C). Indeed, univariate PERMANOVA detected significant differences between sites but no effect of region for these metrics, and no significant effect of the co-variate (Table 3). The biogeographic affinities of species within the assemblages were fairly consistent across the regions, with similar proportions of ‘warm’ and ‘cool’ water species (Fig 4C).

The DISTLM routine was used to determine links between environmental predictor variables and variability in sessile assemblage structure. Marginal tests showed that wave fetch, wave-driven water motion and kelp holdfast age were, individually, the most important predictor variables. The stepwise selection procedure indicated that the most parsimonious model included all environmental variables, which explained 54% of the total observed variability in sessile assemblage structure (Table 4).

**Table 4:** DISTLM marginal test results for each environmental predictor variable selected for the most parsimonious model for sessile assemblages. The best solution based on stepwise selection and AICc criteria is shown. SS = sum of squares (trace), Prop. = proportion of variation explained.

Variable	SS	Pseudo-*F*	P	Prop.
Wave fetch	16127	10.83	0.001	0.134
Wave driven water motion	14886	9.879	0.001	0.124
Kelp age	14268	9.414	0.001	0.119
Holdfast habitable space	10606	6.765	0.001	0.088
Summer temperature	9665	6.112	0.001	0.080
Tidal driven water motion	5489	3.345	0.002	0.046
Summer light intensity	6390	3.925	0.001	0.053
Phosphate concentration	4846	2.937	0.005	0.040
**Best solution: All variables (R^2^: 0.54, AICc: 499.8)**				

### Mobile assemblages

**Fig 6:**
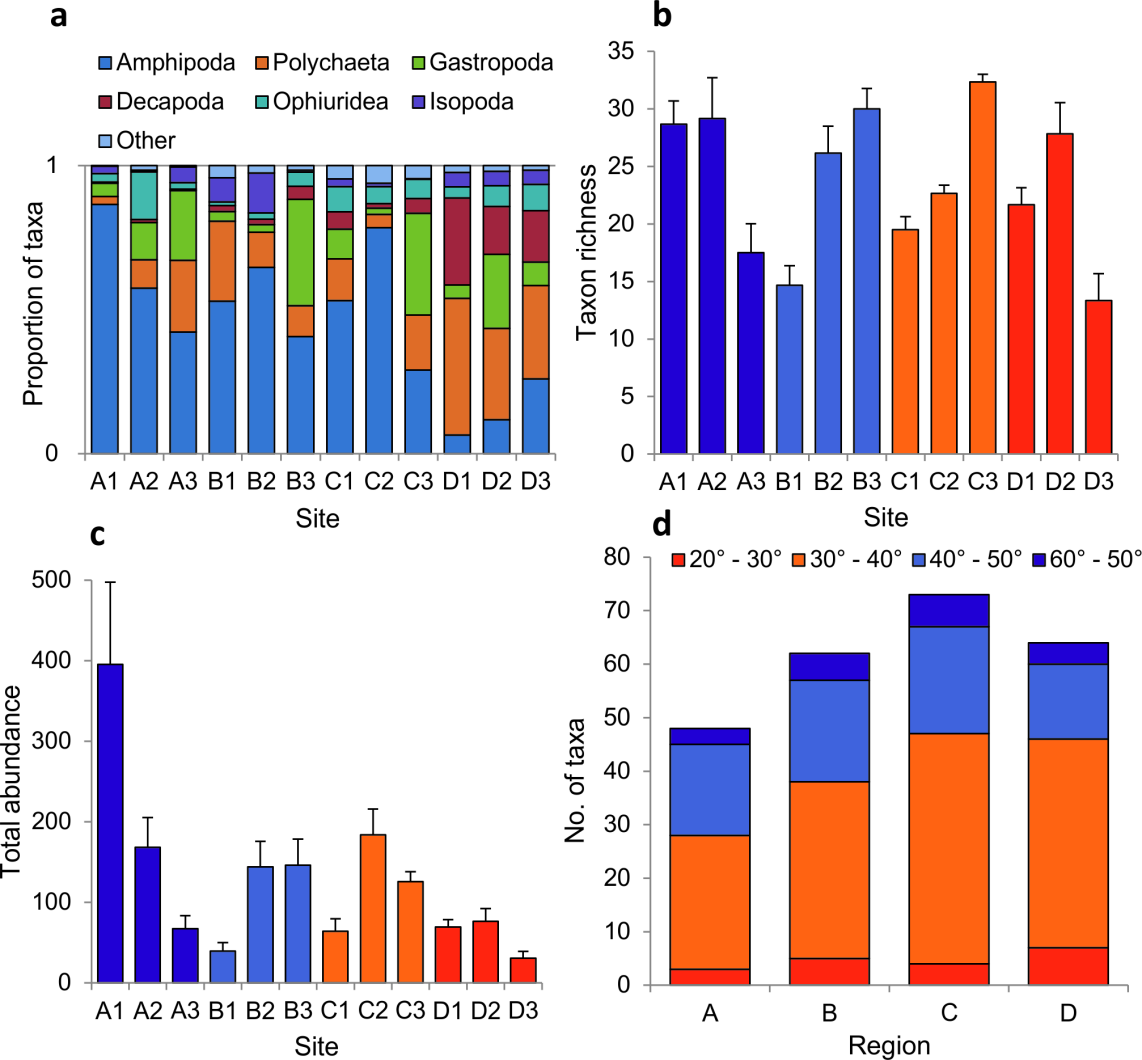
Univariate assemblage-level metrics for mobile holdfast assemblages: (a) the proportion of major taxonomic groups, (b) mobile assemblage taxon richness, (c) total biomass of mobile organisms, (d) taxa equatorward range edge. Values for (b) and (c) are means of 6 replicate holdfasts per site (±SE).

Study-wide, a total of 187 mobile taxa were recorded from 72 holdfasts (S1 Table) and, typically, holdfasts supported an array of mobile taxa (Fig 1). Of all the groups recorded, polychaete worms dominated by richness, making up over a third of the taxa identified (64 taxa). Crustacean groups were also numerous and diverse: gammaridean amphipods (infraorder Gammarida; 37 taxa), Decapoda (order; 13 taxa), Isopoda (order; 11 taxa), Mysida (order; three taxa), Leptostraca (order; one taxon) and Tanaidecea (order; one taxon). Mollusca were also well represented: Gastropoda (class; 34 taxa) and Polyplacophora (class; four taxa). The remainder of the assemblage comprised echinoderms (class Ophiuroidea, class Asteroidea, class Echinoidea, and class Holothuroidea; eight taxa), Pycnogonida (class; four taxa), and three other groups (class Turbellaria, phylum Nemertea, and phylum Sipuncula; four taxa). A sample from southwest England (D1) included a fish (family Gobiesocidae). Mobile fauna were most abundant in northern Scotland (a maximum of >800 individuals per holdfast), which were characterised by high abundances of a few amphipod taxa (namely *Jassa* spp., *Parajassa pelagica* and *Ampithoe* spp.). Study-wide abundances of mobile fauna were, however, highly variable with the lowest value (16 individuals per holdfast) recorded in southwest England. Numerically, amphipods dominated all sites with the notable exception of sites in SW England (D1-D3), which were characterised by high relative abundances of polychaetes and decapod crustaceans (Fig 6A).

Metric MDS plots showed some partitioning between regions, but these patterns were not as clear as those observed in the sessile assemblage data (Fig 5C). Similarly, while the mMDS plots showed some partitioning between sites, within regions, this is less evident than those observed for sessile assemblages; generally, there is considerably more variation in mobile assemblages, both within regions, and within sites. Interestingly, there was some evidence of partitioning between the two southern regions (C & D) and the two northern regions (A & B), a pattern not observed for sessile assemblages (Fig 5D). PERMANOVA identified significant differences between regions and sites, as well as a significant effect of the co-variate (Table 3). Pairwise tests within the region factor showed that assemblages in southwest England were statistically distinct from those in other regions. PERMDISP did not detect any significant differences in multivariate dispersion between regions (F_3,68_ = 0.92, P = 0.486).

SIMPER analysis showed that the observed differences between regions were driven primarily by a markedly lower abundance of the amphipod *Jassa* spp. within southwest England compared to the other regions (S4 and S5 Tables). The abundance of other amphipods (e.g. *Lysianassa certatina*, *Ampithoe* spp., *L*e*mbos websteri*, *Erichthonius* sp., *Caprella* spp.) was notably lower in southwest England compared with the other regions, and was a major contributor to the observed dissimilarity between regions. Samples from southwest England were characterised by high abundances of the crab *Pisidia longicornis* and the sabellid polychaete *Branchiomma bombyx* compared to all other regions (S4 and S5 Tables).

Taxon richness varied markedly between sites within regions, with values across regions being comparable (Fig 6B). Total abundance varied by a factor of ~8 within a single region (north Scotland), with the lowest values recorded at sites in southwest England (Fig 6C). For both metrics, we recorded a significant effect of site and the co-variate, but no overall effect of region (Table 3). As with the sessile assemblage, the biogeographic affinities of species within the assemblages was fairly consistent across the regions, with similar proportions of ‘warm’ and ‘cool’ water species (Fig 6D).

The DISTLM routine showed that, based on marginal tests, summer temperature, wave fetch and summer light intensity were the most important predictors of mobile assemblage structure (Table 5). The stepwise selection procedure indicated that the most parsimonious solution, which explained 47% of the total observed variability in assemblage structure, included all the predictor variables (Table 5).

**Table 5:** DISTLM marginal test results for each environmental predictor variable selected for the most parsimonious model for mobile assemblages. The best solution based on stepwise selection and AICc criteria is shown. SS = sum of squares (trace), Prop. = proportion of variation explained.

Variable	SS	Pseudo-*F*	P	Prop.
Summer temperature	21147	9.299	0.001	0.117
Wave fetch	15245	6.464	0.001	0.085
Summer light intensity	14745	6.234	0.001	0.082
Holdfast habitable space	12673	5.291	0.001	0.070
Kelp age	10553	4.351	0.001	0.059
Tidal driven water motion	9813	4.029	0.001	0.054
Wave driven water motion	8477	3.453	0.001	0.047
Phosphate concentration	6525	2.629	0.003	0.036
**Best solution: All variables (R^2^: 0.47, AICc: 539.0)**				

### Relationships between sessile and mobile assemblages

Spatial variability patterns of sessile and mobile assemblages were not well correlated. Similarity matrices generated at the holdfast level were very weakly correlated (ρ = 0.27, P = 0.01), whereas matrices generated at the site (ρ = 0.21, P = 0.07) and region (ρ = 0.04, P = 0.50) level were not correlated, indicating that sessile and mobile assemblages exhibited distinct variability patterns at all spatial scales. Similarly, there was no correlation in taxon richness (r = -0.19, P = 0.15, S1 Fig) or total biomass/abundance (r = -0.05, P = 0.68, S1 Fig) between sessile and mobile assemblages.

## Discussion

Kelp holdfasts are important repositories of biodiversity in coastal marine ecosystems. The assemblages sampled in this study were highly diverse, in some cases highly abundant, and fairly typical of *Laminaria hyperborea* holdfasts described in previous research [2, 45, 56, 75]. We recorded a study-wide richness value of 261 distinct taxa, while average richness per holdfast was ~40 taxa (i.e. combined averages for sessile and mobile richness). These values are comparable, but notably higher, than most previous reports of holdfast richness, with the exception of *Ecklonia radiata* holdfasts in Australasia and *L*. *hyperborea* holdfasts in Norway (see [38] for a comprehensive review). These values are also comparable to study-wide richness values for macroinvertebrate assemblages associated with seagrass beds in both the Mediterranean and Western Australia [76, 77], which are widely regarded as habitats with high biodiversity value [78]. With regards to faunal densities, average mobile invertebrate abundance exceeded ~400 individuals per holdfast at one study site but was typically >150 individuals per holdfast, while mean biomass of sessile assemblages was typically ~10 g per holdfast. Given that densities of mature *L*. *hyperborea* plants are in the order of 10 plants m^-2^ at these study sites [58], a simple approach to ‘scaling up’ would yield estimates of faunal abundance of 1500 inds.m^-2^ and faunal biomass of 100 g m^-2^ for holdfast assemblages alone. As *L*. *hyperborea* populations in the UK are predicted to inhabit an area in the region of 8,100 [58] to 16,000 km^2^ [64], holdfast associated assemblages are likely to play a significant role in trophic processes and wider coastal ecosystem functioning.

We recorded marked between-region and between-site variability in the structure of both sessile and mobile macrofaunal assemblages. In general, mobile assemblages were more diverse than their sessile equivalents, and tended to be more heterogeneous and spatially variable. Although we observed considerable regional-scale variability, we did not record predictable shifts in assemblage diversity or structure with latitude. That is, differences between regions did not generally correspond with sequential shifts in latitude or temperature. For sessile taxa, holdfast assemblages in north Scotland, west Scotland and southwest England were all distinct from one another, whereas assemblages from Wales were far more heterogeneous and were not dissimilar to other regions. For mobile assemblages, there was some indication of latitudinal shifts in structure (see below) and, despite high site-level variability, assemblages in southwest England were statistically distinct from those in other regions. Evidence of regional-scale variability contrasts with a comparable study on *E*. *radiata* holdfasts in New Zealand [66], which found holdfast assemblages structure to be relatively consistent and predictable along a gradient of 2° in latitude and >300 km of coastline. As our study encompassed a larger spatial gradient, it is likely that between-region variability in key drivers of ecological pattern such as temperature, wave exposure and primary productivity in the overlying water column was more influential.

For example, while wave fetch and local water motion were broadly comparable between regions, overall wave exposure values were slightly greater in north Scotland and southwest England compared to west Scotland and southwest Wales (except C1). *L*. *hyperborea*, like many other kelp species, exhibits morphological adaptations to hydrodynamic forcing, including the development of larger holdfasts under wave exposed conditions [79], which was perhaps reflected in the generally larger holdfasts in north Scotland and (to a lesser extent) southwest England. The size and shape of the holdfast habitat can be an important driver of diversity and abundance of associated assemblages in some systems [41, 51, 56], although this relationship has been shown to break down elsewhere [2, 80], and may be more important for smaller, younger holdfasts [39, 66]. Here, differences in holdfast structure may also be driven, at least in part, by regional-scale variability in biogenic habitat structure. Other possible drivers of variability operating across regional scales include patterns of dispersal and connectivity, coastal geomorphology and differences in proximal habitat types and potential source populations.

Similarly, regional scale variability in turbidity, sedimentation and the supply of organic matter may be important in determining holdfast assemblage structure. In this case, holdfasts from southwest England were largely distinct from those elsewhere, primarily because of markedly lower abundances of amphipods, and higher relative abundances of crabs, polychaetes, and sponges. The marine environments around Wales (i.e. the Irish Sea) and West Scotland (i.e. the Firth of Lorne) are typically more turbid, with higher levels of suspended material [41, 58], and holdfasts here were characterised by high silt deposition. A considerable proportion of the mobile assemblages at these sites was composed of filter and deposit feeding amphipods (e.g. *Jassa* spp., *Monocorophium sextonae*), which would conceivably benefit from high levels of particulate organic matter. In contrast, the less turbid waters in southwest England could favour different taxa, and could explain a lower dominance of amphipod deposit feeders and a higher dominance of omnivorous crabs. As such, regional-scale differences in turbidity or the deposition of organic matter from riverine inputs or pelagic primary production may influence the development of holdfast assemblages and trophic structuring [25, 51]. Unfortunately, we did not directly measure the sediment content of each holdfast, which would have been a useful predictor variable given that previous research has shown correlations between the abundances of taxa and sediment loads [51]. Given that coastal development and maritime activities (e.g. dredging) can alter sedimentation rates, further investigation into the influence of sediment content on holdfast assemblages would be useful.

The structure of mobile assemblages did exhibit some latitudinal patterns, with some distinction in multivariate structure between northernmost (north and west Scotland) and southernmost (southwest Wales and southwest England) sites, and sea temperature emerging as an important explanatory variable. This was largely driven by a general increase in the relative abundance of amphipods from south to north and, in contrast, an increase in the relative abundance of decapods and polychaetes from north to south. Conversely, sessile species were widely distributed across the regions and sessile assemblages were more influenced by wave exposure and local water motion. Such differences in variability patterns between mobile and sessile fauna may be linked to dispersal potential. The most abundant mobile fauna, in this instance gammaridean amphipods, tend to brood their offspring and may have relatively limited dispersal potential compared to many commonly found sessile fauna, which have a planktonic larval stage [81–84]. Moreover, recent work has shown that the structure of kelp forests varies with latitude along the gradient encapsulated in this study [19] and, as different kelp species may harbour distinct associated assemblages [47, 85–87], the composition of the wider kelp forest may potentially play a role in the development of distinct, regional holdfast assemblages. This may be particularly important for mobile assemblages, as some common taxa (e.g. amphipods and isopods) have been shown to move freely throughout kelp forests and even to emigrate to adjacent systems [75, 88]. As such, the structure and configuration of surrounding habitats, and their associated species pools, is likely to play an important role in the structuring of holdfast assemblages and may vary somewhat predictably with latitude.

Pronounced site-level variability was an important component of the observed spatial variation in holdfast community structure and diversity, suggesting that environmental factors varying across similar spatial scales, such as wave exposure, could be important in determining ecological pattern. Previous research has highlighted the importance of hydrodynamic forces (i.e. the action of waves and the tide) in structuring marine communities [23, 24, 89], including kelp systems [22, 79, 90–92], and in relation to kelp-associated assemblages in particular [21, 93, 94]. The effect of wave exposure on the morphology of *L*. *hyperborea* is well documented (e.g. the development of larger holdfasts [79]), and as faunal diversity is often (but not always) related to habitat size, a relationship exists between wave exposure and kelp associated faunal diversities [2, 21, 66, 80, 93–97]. Intense wave action represents a physical disturbance to algal associated fauna, and may result in considerable loss of fauna due to dislodgement and mortality [49, 50]. The reduced diversity and abundances of communities associated with holdfasts from the most wave exposed sites (i.e. A1) in this study suggest this process may play a role within kelp holdfasts. The largest holdfasts, by some margin, were found at site A1, the most exposed site in north Scotland. While the mobile assemblage associated with these holdfasts was diverse and abundant, as one would expect, the sessile assemblage was comparatively depauperate in relation to the apparent habitable space available. Holdfasts from this site were missing the high abundances and biomass of common bryozoan species in particular, and were characterised by a high sediment load made of coarse sand, and had an almost ‘sand-blasted’ look. It is likely that the strong wave motion characteristic of this site, coupled with the large, open nature of the holdfasts, caused smothering of delicate filter-feeding organisms, such as the bryozoans so characteristic of other sites.

Interestingly, the potential effects of wave exposure seemed to be more pronounced on sessile, rather than on mobile assemblages. This impact is potentially due to the influence of sedimentation, which can smother delicate filter-feeding sessile organisms [25] but may have exert a positive influence over other taxa [51]. Site-level variability may also be promoted by differences in reef structure and substratum characteristics. Topographic complexity, the prevalence of large boulders versus pavement-like platforms, and reef rugosity may influence kelp population structure [98–100]. As such, variation in holdfast morphology (i.e. relative holdfast volume) driven by site-level differences in reef structure may have influenced the development and richness of associated assemblages. Other factors that have been identified as important drivers of site-level variability in holdfast assemblages include pollution [101], kelp harvesting and farming [80, 102] and grazing pressure [103, 104]. However, given the negligible impact of these activities and processes across the current study area [8], they were unlikely to be important in this case.

In addition to the variability between regions and between sites, we recorded pronounced small-scale variability between individual holdfasts separated by a few meters. Marine benthic communities generally exhibit considerable small-scale variability [105, 106], which is driven by a range of processes operating across multiple spatial, and temporal scales [61]. Combined with the highly variable nature of supply-side ecology [107], the inter-holdfast variability recorded here is perhaps not surprising. Small-scale variations in food availability, protection from predation, water movement and sedimentation (e.g. due to proximity to topographical reef features which may attenuate wave action), and differences in species’ tolerance to smothering or dislodgement may also influence assemblage structure [61, 108, 109]. Moreover, the structure of the kelp forest itself can induce small-scale variability in associated assemblages; kelp plant density, the wider habitat matrix, the level of patchiness and whether the kelp canopy is monospecific or mixed have been shown to influence local diversity patterns [110–112]. Indeed, much of the variability observed in holdfast community structure throughout this study was unexplained by the environmental factors measured. It is likely that these factors were measured at a spatial scale too large to account for within-site variation, highlighting the need for more research into the small, local scale drivers of holdfast assemblage development and maintenance.

In conclusion, faunal assemblages associated with *L*. *hyperborea* holdfasts are highly diverse and exhibit considerable structural variability over multiple spatial scales. Crucially, the sessile and mobile components of holdfast assemblages exhibited different patterns and may be influenced by key environmental drivers, specifically variability in wave exposure and temperature, to differing degrees due to divergence in life histories or growth strategy. It is evident that *L*. *hyperborea* serves as a critical foundation species in shallow rocky habitats in the northeast Atlantic by providing biogenic habitat, altering environmental conditions and exerting a strong influence over local biodiversity and community structure. Within UK waters, our study suggests that local scale environmental variability is more important in structuring kelp-associated assemblages than latitudinal-scale variation in sea temperature. However, given that sea water temperatures around the UK have significantly increased in recent decades [113], and are predicted to continue to rise through the next century [114], it is likely that temperature will begin to play a larger role in structuring the biogenic habitat provided by *L*. *hyperborea*, and thereby its associated assemblages, in the near future. The ecophysiology of *L*. *hyperborea* is adversely impacted by temperatures above 20°C [115], and as the southernmost regions of the UK currently experience temperatures of this magnitude during anomalous warming events [116] and are projected to experience such temperatures more frequently in the coming decades [114], the continued provision of biogenic habitat is at risk from future climate change. Any climate-driven reduction in the biomass, density, spatial extent or longevity of *L*. *hyperborea* will likely result in a reduction of habitat available for colonisation and consequent changes to community structure and local biodiversity [87]. Indeed, further south on the Iberian Peninsula, *L*. *hyperborea* has retracted its equatorward range edge as marginal populations have responded to ocean warming [20, 60], and key ecological functions including habitat provision and benthic primary productivity have been lost or altered. Changes in habitat provision will likely influence holdfast assemblage structure, and in doing so, affect the usefulness of kelp holdfasts for biodiversity monitoring and detecting local environmental impacts [117]. Clearly, a better understanding of the drivers of kelp community structure, including an improved appreciation of species interactions in a rapidly changing environment, is required to predict the structure and conserve the diversity of these ecosystems in the coming decades.

## Supporting information

S1 Table Complete list of taxa identified, including higher taxonomic groups. Crosses denote in which regions examples of each taxa were recorded. Sessile assemblage precedes mobile assemblage.
(DOCX)

S2 Table Percentage contributions of individual taxa to observed differences in sessile holdfast assemblages between regions, as determined by SIMPER analysis. Biomass values were fourth-root transformed and standardised by habitable holdfast space prior to analysis.
(DOCX)

S3 Table Mean biomass values (± SE) for taxa that contributed most to the observed dissimilarities in sessile assemblage structure between regions, as determined by SIMPER (see S2 Table). (DOCX)

S4 Table Percentage contributions of individual taxa to observed differences in mobile holdfast assemblages between regions, as determined by SIMPER analysis. Abundance values were fourth-root transformed and standardised by habitable holdfast space prior to analysis.
(DOCX)

S5 Table Mean abundance values (± SE) for taxa that contributed most to the observed dissimilarities in mobile assemblage structure between regions, as determined by SIMPER (see S4 Table). (DOCX)

S1 Fig **Scatterplots of (a) taxon richness and (b) total biomass/abundance of sessile versus mobile assemblages**. Each data point represents a single holdfast sample (north Scotland = dark blue; west Scotland = light blue; west Wales = pink; southwest England = dark red).
(DOCX)
