# Supplementary material for: Spatial variability in the diversity and structure of faunal assemblages associated with kelp holdfasts (Laminaria hyperborea) in the northeast Atlantic
Source: PLoS One. 2018 Jul 12;13(7):e0200411. doi: 10.1371/journal.pone.0200411 (PMC6042752; doi:10.1371/journal.pone.0200411)
Supplement: S1 Table — Crosses denote in which regions examples of each taxa were recorded. Sessile assemblage precedes mobile assemblage. (DOCX) [file pone.0200411.s001.docx]

| **S1 Table. Complete list of taxa identified, including higher taxonomic groups**. Crosses denote in which regions examples of each taxa were recorded. Sessile assemblage precedes mobile assemblage. | | | | | |
| --- | --- | --- | --- | --- | --- |
| Group | **Species** | **A** | **B** | **C** | **D** |
| Anthozoa | *Actiniidae spp.* | X |  | X |  |
| Ascidiacea | *Ascidan spp.* | X | X | X | X |
|  | *Didemnid* | X | X | X | X |
| Bivalvia | *Anomia ephippium* | X | X | X | X |
|  | *Hiatella arctica* | X | X | X | X |
|  | *Kellia suborbicularis* | X |  |  | X |
|  | *Mediolula phaseolina* |  |  |  |  |
|  | *Mimachlamys varia* | X | X | X |  |
|  | *Musculus subpictus* | X | X | X | X |
|  | *Mytilus spp.* | X | X | X | X |
|  | *Parvicardium spp.* |  | X |  |  |
|  | *Pectinidae spp.* |  |  | X |  |
|  | *Tapes rhomboides* | X |  |  |  |
|  | *Timoclea ovata* | X |  |  |  |
|  | *Turtonia minuta* | X |  |  |  |
|  | *Venerupis corrugata* | X | X |  |  |
| Bryozoa | *Aetea anguina* |  | X |  |  |
|  | *Amathia lendigera* |  |  |  | X |
|  | *Amphiblestrum flemingii* |  | X |  |  |
|  | *Bicellariella ciliata* |  | X |  |  |
|  | *?Bugulina fulva* |  |  | X |  |
|  | *Callopora lineata* | X | X | X | X |
|  | *Callopora rylandi* | X |  |  |  |
|  | *Cellepora pumicosa* |  |  |  | X |
|  | *Celleporella hyalina* | X | X | X | X |
|  | *Celleporina caliciformis* | X | X | X | X |
|  | *Cradoscrupocellaria reptans* | X | X | X | X |
|  | *Crisia aculeata* |  |  | X |  |
|  | *Crisia denticulata* | X | X | X | X |
|  | *Crisia eburnea* |  | X | X | X |
|  | *Crisida cornuta* | X | X | X | X |
|  | *Disporella spp.* | X | X | X | X |
|  | *Electra pilsoa* | X | X | X | X |
|  | *?Escharella labisoa* |  |  | X | X |
|  | *Escharoides coccinea* | X | X | X | X |
|  | *Fenestrulina malusii* |  | X | X |  |
|  | *Filicrisia geniculata* | X |  | X | X |
|  | *Membranipora membranacea* | X |  |  |  |
|  | *Membraniporella nitida* | X |  |  |  |
|  | *Microporella ciliata* | X | X | X | X |
|  | *Omalosecosa ramulosa* |  |  |  | X |
| Bryozoa | *Oshurkovia (Umbonula) littoralis* | X |  | X |  |
|  | *Phaeostachys spinifera* |  | X |  |  |
|  | *Schizomavella hastata* |  |  |  | X |
|  | *Schizomavella linearis* |  | X |  | X |
|  | *Scruparia chelata* |  | X |  | X |
|  | *Scrupocellaria scrupea* |  |  | X | X |
|  | *Scrupocellaria scruposa* | X | X | X | X |
|  | *Smittina affinis* |  |  |  | X |
|  | *Tubulipora spp.* |  | X | X | X |
| Cirripedia | *Acasta spongites* | X |  |  | X |
|  | *Balanus balanus* | X |  | X |  |
|  | *Balanus crenatus* |  | X | X | X |
|  | *Chirona hameri* |  |  | X |  |
|  | *Hesperibalanus fallax* |  |  |  | X |
|  | *Verruca stroemia* | X | X | X | X |
| Hydrozoa | *Abietinaria filicula* | X |  |  |  |
|  | *Amphisbetia operculata* |  |  | X |  |
|  | *Diphasia attenuata* |  | X | X |  |
|  | *Kirchenpaueria pinnata* |  |  |  | X |
|  | *Obelia spp.* | X | X | X | X |
|  | *Plumularia setacea* |  |  | X | X |
|  | *Sertularella spp.* |  |  | X | X |
|  | *Sertularia distans* |  |  |  | X |
| Polychaeta | *Pomatoceros spp.* | X | X | X | X |
|  | *Serpulid spp.* |  | X | X | X |
|  | *Spirobid spp.* | X | X | X | X |
| Porifera | *Demosponge A* | X |  | X | X |
|  | *Demosponge B* |  |  |  | X |
|  | *Demosponge D* | X | X |  |  |
|  | *Demosponge E* |  |  |  | X |
|  | *Demosponge F* | X |  |  | X |
|  | *Demosponge G* | X |  |  | X |
|  | *Dysidea fragilis* |  |  |  | X |
|  | *Flat sponge* |  | X | X | X |
|  | *Sycon ciliata* | X | X |  | X |
| Actinopterygii | *Gobiesocidae spp.* |  |  |  | X |
| Amphipoda | *?Gitana sarsi* | X |  |  |  |
|  | *Abludomelita obtusata* | X |  |  |  |
|  | *Acanthonotozomatidae spp.* | X | X | X | X |
|  | *Amphilochus manudens* |  | X | X |  |
|  | *Ampithoe spp.* | X | X | X |  |
|  | *Aora typica* | X |  |  | X |
|  | *Aoridae ♀* | X | X | X | X |
|  | *Apherusa bispinosa* | X | X |  | X |
|  | *Apherusa jurinei* | X | X | X |  |
| Amphipoda | *Apolochus neapolitanus* | X |  |  |  |
|  | *Caprella acanthifera* | X | X | X |  |
|  | *Caprella septentrionalis* |  | X |  |  |
|  | *Caprella sp. A* |  | X | X | X |
|  | *Caprella spp. complex* | X | X | X |  |
|  | *Dexamine spinosa* | X | X | X | X |
|  | *Dexamine thea* |  | X |  |  |
|  | *Elasmopus rapax* |  |  |  | X |
|  | *Erichthonius* sp. | X | X | X | X |
|  | *Gammaropsis maculata* | X |  | X | X |
|  | *Gitana sarsi* |  | X |  |  |
|  | *Jassa spp.* | X | X | X | X |
|  | *Lembos websteri* | X | X | X | X |
|  | *Leucothoe spinicarpa* | X |  | X | X |
|  | *Liljeborgia pallida* |  |  | X |  |
|  | *Lysianassa certatina* | X | X | X | X |
|  | *Maera grossimana* |  |  |  | X |
|  | *Melitidae spp.* |  |  |  | X |
|  | *Monocorophium sextonae* | X | X | X | X |
|  | *Orchomene spp.* | X |  |  |  |
|  | *Othomaera othonis* | X |  |  |  |
|  | *Parajassa pelagica* | X |  | X |  |
|  | *Pereionotus testudo* |  |  |  | X |
|  | *Phtisica marina* | X | X | X | X |
|  | *Pseudoprotella phasma* |  |  | X |  |
|  | *Stenothoidae spp.* | X | X |  |  |
|  | *Tritaeta gibbosa* | X | X | X | X |
|  | *Tryphosella sarsi* | X | X | X |  |
| Asteroidea | *Asterias rubens* | X |  | X |  |
|  | *Asterina gibbosa* |  |  |  | X |
|  | *Asteroidea juv.* | X | X | X |  |
| Decapoda | *Cancer pagurus* | X | X |  |  |
|  | *Decapoda juv.* | X | X |  |  |
|  | *Eualus occultus* |  |  |  | X |
|  | *Eualus pusiolus* | X | X | X |  |
|  | *Eualus spp.* |  | X |  |  |
|  | *Eurynome spinosa* | X |  | X | X |
|  | *Eurynome spp.* | X |  |  |  |
|  | *Galathea spp.* | X | X | X | X |
|  | *Galathea strigosa* |  |  |  | X |
|  | *Hippolyte varians* |  | X | X |  |
|  | *Hippolytidae spp.* |  | X |  |  |
|  | *Pilumnus hirtellus* | X | X | X | X |
|  | *Pisidia longicornis* | X | X | X | X |
| Echinoidea | *Psammechinus miliaris* | X | X | X |  |
| Gastropoda | *?Buccinidae spp.* | X | X | X |  |
| Gastropoda | *?Manzonia zetlandica* | X |  |  |  |
|  | *Calliostoma ziziphinum* |  |  |  | X |
|  | *Cerithiopsis spp.* |  |  |  | X |
|  | *Cerithiopsis tubercularis/barleei* | X |  |  | X |
|  | *Chauvetia brunnea* |  |  |  | X |
|  | *Crisilla semistriata* | X | X | X | X |
|  | *Fissurellidae spp.* |  |  |  | X |
|  | *Gibbula cineraria* | X | X | X | X |
|  | *Lacuna parva* | X |  |  |  |
|  | *Lacuna vincta* | X | X | X |  |
|  | *Lamellaria perspicua/latens* |  |  |  | X |
|  | *Margarites helicinus* | X | X |  |  |
|  | *Marshallora adversa* |  |  |  | X |
|  | *Nudibranchia spp.* | X |  |  |  |
|  | *Ocenebra erinaceus* |  | X |  |  |
|  | *Onchidorididae spp.* |  | X | X |  |
|  | *Onoba aculeus* | X |  |  |  |
|  | *Onoba semicostata* | X | X |  | X |
|  | *Onoba spp.* | X | X | X |  |
|  | *Opisthobracnchia spp.* |  | X |  |  |
|  | *Patella pellucida* | X | X | X | X |
|  | *Retusa truncatula* | X |  |  |  |
|  | *Rissoa lilacina* | X |  |  |  |
|  | *Rissoa parva (Rissoidae sp A)* | X | X | X | X |
|  | *Rissoaidae sp. B (smooth)* | X | X | X |  |
|  | *Rissoidae sp. C* | X | X | X |  |
|  | *Spiralinella spiralis* |  | X |  |  |
|  | *Tectura virginea* | X | X |  |  |
|  | *Tricolia pullus* |  | X | X | X |
|  | *Tritia incrassata* | X |  | X | X |
|  | *Trivia arctica* |  |  | X | X |
|  | *Trivia monacha* |  |  |  | X |
|  | *Velutina velutina* |  |  | X |  |
| Holothuroidea | *Cucumariidae spp.* |  |  | X | X |
| Isopoda | *?Idotea neglecta* |  |  | X |  |
|  | *Anthura gracilis* |  | X |  |  |
|  | *Astacilla damnoniensis* |  |  |  | X |
|  | *Cymodoce truncata* |  |  |  | X |
|  | *Idotea gramulosa* | X |  |  |  |
|  | *Idotea pelagica* | X |  |  |  |
|  | *Jaeropsis brevicornis brevicornis* |  |  |  | X |
|  | *Janira maculosa* | X | X | X | X |
|  | *Janiropsis breviremis* | X |  |  |  |
|  | *Munna kroyeri* | X | X |  |  |
|  | *Stenosoma lancifer* |  |  |  | X |
| Leptostraca | *Nebalia bipes* |  | X |  |  |
| Mysida | *Heteromysis norvegica* |  |  | X |  |
|  | *Mysidae spp.* | X |  |  |  |
|  | *Praunus inermis* |  | X |  | X |
| Ophiuroidea | *Amphipholis squamata* | X | X | X | X |
|  | *Ophiothrix fragilis* | X | X | X | X |
|  | *Ophiurida spp.* | X |  |  |  |
| Polychaeta | *?Eupolymnia/Neoamphitrite spp.* | X |  |  |  |
|  | *?Glycera capitata* |  | X |  |  |
|  | *?Harmothoe imbricata* |  | X |  |  |
|  | *?Oriopsis spp.* |  |  |  | X |
|  | *?Paraonidae spp.* | X |  |  |  |
|  | *?Proceraea scapularis* |  | X |  |  |
|  | *?Spio goniocephala* | X |  |  |  |
|  | *?Trypanosyllis spp.* |  |  | X |  |
|  | *Alentia gelatinosa* |  | X |  |  |
|  | *Amblyosyllis formosa* |  | X | X | X |
|  | *Aonides oxycephala* | X | X | X |  |
|  | *Branchiomma bombyx* |  |  |  | X |
|  | *Capitella capitata* |  |  | X |  |
|  | *Cirratulidae spp.* | X |  |  |  |
|  | *Eulalia spp.* | X | X | X |  |
|  | *Eulalia viridis* | X | X | X | X |
|  | *Eumida spp.* | X | X |  | X |
|  | *Eunice spp.* |  |  |  | X |
|  | *Eurysyllis tuberculata* |  |  |  | X |
|  | *Eusyllis blomstrandi* | X | X |  | X |
|  | *Flabelligera affinis* |  |  | X |  |
|  | *Glycera spp.* | X |  |  |  |
|  | *Harmothoe extenuata* | X | X | X | X |
|  | *Harmothoe impar* | X | X | X | X |
|  | *Harmothoe pagenstecheri* | X | X | X | X |
|  | *Harmothoe spinifera* | X |  | X | X |
|  | *Harmothoe spp.* | X | X | X | X |
|  | *Jasmineria elegans* |  |  |  | X |
|  | *Lepidonotus clava* | X |  | X | X |
|  | *Lepidonotus squamatus* | X | X | X |  |
|  | *Lysidice ninetta* |  |  | X | X |
|  | *Lysidice unicornis* |  |  |  | X |
|  | *Malacoceros spp.* | X | X | X |  |
|  | *Maldanidae spp.* | X |  |  |  |
|  | *Myrianida spp.* | X | X |  |  |
|  | *Nemertea spp.* | X | X | X | X |
|  | *Nereimyra punctata* | X | X | X |  |
|  | *Nereis pelagica* | X | X | X | X |
|  | *Nicolea venustula* |  | X | X | X |
|  | *Odontosyllis spp.* | X | X | X | X |
| Polychaeta | *Perinereis cultrifera* |  |  | X |  |
|  | *Perkinsiana spp.* |  |  |  | X |
|  | *Pherusa plumosa* |  |  | X |  |
|  | *Pholoe baltica (sensu Barnich 211)* |  |  | X |  |
|  | *Pholoe inornata (sensu Barnich 211)* | X | X | X | X |
|  | *Phyllodoce greenlandia/laminacea* |  |  | X |  |
|  | *Phyllodoce maculata* |  | X | X |  |
|  | *Platynereis dumerilii* |  | X | X | X |
|  | *Poecilochaetus serpens* |  |  | X |  |
|  | *Polycirrus spp.* | X | X |  | X |
|  | *Polydorid spp.* |  |  |  | X |
|  | *Polynaidae (Malnigrenia?)* |  |  | X |  |
|  | *Proceraea picta* |  | X | X | X |
|  | *Proceraea spp.* |  |  | X | X |
|  | *Psamathe fusca* | X | X | X |  |
|  | *Sabellaria spinulosa* | X |  | X | X |
|  | *Sclerocheilus minutus* |  |  |  | X |
|  | *Spionidae sp. A* | X |  |  |  |
|  | *Sphaerodoridae spp.* | X |  |  |  |
|  | *Sphaerodorum gracilis* |  | X | X |  |
|  | *Sthenelais boa* | X |  |  |  |
|  | *Syllis spp.* | X | X | X | X |
|  | *Terebellidae spp.* |  |  | X |  |
|  | *Trypanosyllis spp.* |  |  | X | X |
|  | *Websterinereis glauca* |  |  | X | X |
| Polyplacophora | *?Tonicella rubra/Lepidochitona cancreas* | X |  |  |  |
|  | *Acanthochitona crinita* | X | X | X | X |
|  | *Ischnochitonidae spp.* |  | X |  |  |
|  | *Polyplacophora juv.* |  | X |  |  |
| Pycnogonida | *Achelia echinata* | X | X | X |  |
|  | *Ammothella longioculata* |  |  | X |  |
|  | *Callipallene tiberi* |  |  | X |  |
|  | *Endeis spinosa* | X | X | X |  |
| Sipuncula | *Sipuncula spp.* | X | X | X |  |
| Tanaidacea | *Apseudes talpa* |  |  | X |  |
| Turbellaria | *Platyhelminthes (Turbellaria)* |  |  | X |  |
|  | *Turbellaria spp.* |  | X |  |  |
