# Supplementary material for: Spatial variability in the diversity and structure of faunal assemblages associated with kelp holdfasts (Laminaria hyperborea) in the northeast Atlantic
Source: PLoS One. 2018 Jul 12;13(7):e0200411. doi: 10.1371/journal.pone.0200411 (PMC6042752; doi:10.1371/journal.pone.0200411)
Supplement: S2 Table — Biomass values were fourth-root transformed and standardised by habitable holdfast space prior to analysis. (DOCX) [file pone.0200411.s002.docx]

| **S2 Table. Percentage contributions of individual taxa to observed differences in sessile holdfast assemblages between regions, as determined by SIMPER analysis.** Biomass values were fourth-root transformed and standardised by habitable holdfast space prior to analysis. | | | | | | |
| --- | --- | --- | --- | --- | --- | --- |
| Species | Av. abund | Av. abund | Av. diss | Diss/SD | Contrib% | Cum% |
|  | **N Scot (A)** | **W Scot (B)** |  | | | |
| *Spirobranchus* spp. | 0.14 | 0.29 | 3.82 | 1.34 | 6.29 | 6.29 |
| *Celleporina hassallii* | 0.19 | 0.02 | 3.78 | 3.43 | 6.22 | 12.51 |
| *Balanus crenatus* | 0.00 | 0.13 | 3.08 | 0.68 | 5.08 | 17.59 |
| *Crisidia cornuta* | 0.03 | 0.14 | 2.80 | 1.33 | 4.61 | 22.20 |
| *Verruca stroemia* | 0.18 | 0.07 | 2.76 | 1.63 | 4.55 | 26.75 |
|  | **N Scot (A)** | **SW Eng (D)** |  | | | |
| Demosponge A | 0.05 | 0.23 | 3.75 | 1.69 | 7.31 | 7.31 |
| *Mytilus* spp. | 0.16 | 0.02 | 2.81 | 2.45 | 5.48 | 12.79 |
| *Didemnidae* spp. | 0.10 | 0.21 | 2.48 | 1.57 | 4.83 | 17.62 |
| Demosponge F | 0.01 | 0.13 | 2.42 | 1.25 | 4.71 | 22.33 |
| *Verruca stroemia* | 0.18 | 0.29 | 2.38 | 1.17 | 4.64 | 26.97 |
|  | **W Scot (B)** | **SW Eng (D)** |  | | | |
| *Verruca stroemia* | 0.07 | 0.29 | 3.98 | 2.21 | 6.71 | 6.71 |
| Demosponge A | 0.00 | 0.23 | 3.89 | 2.02 | 6.56 | 13.27 |
| *Celleporina hassallii* | 0.02 | 0.18 | 3.00 | 2.06 | 5.07 | 18.34 |
| *Didemnidae* spp. | 0.06 | 0.21 | 2.88 | 1.75 | 4.87 | 23.21 |
| *Balanus crenatus* | 0.13 | 0.02 | 2.45 | 0.74 | 4.14 | 27.35 |
