# Supplementary material for: Spatial variability in the diversity and structure of faunal assemblages associated with kelp holdfasts (Laminaria hyperborea) in the northeast Atlantic
Source: PLoS One. 2018 Jul 12;13(7):e0200411. doi: 10.1371/journal.pone.0200411 (PMC6042752; doi:10.1371/journal.pone.0200411)
Supplement: S3 Table — (DOCX) [file pone.0200411.s003.docx]

| **S3 Table. Mean biomass values (± SE) for taxa that contributed most to the observed dissimilarities in sessile assemblage structure between regions, as determined by SIMPER (see S2 Table).** | | | |
| --- | --- | --- | --- |
|  | **N Scot (A)** | **W Scot (B)** |  |
| *Spirobranchus* spp. | 0.30 ± 0.08 | 1.45 ± 0.41 |  |
| *Celleporina caliciformis* | 0.55 ± 0.13 | 0.00 ± 0.00 |  |
| *Balanus crenatus* | 0.00 ± 0.00 | 1.17 ± 0.64 |  |
| *Crisidia cornuta* | 0.04 ± 0.02 | 0.24 ± 0.08 |  |
| *Verruca stroemia* | 0.68 ± 0.22 | 0.02 ± 0.01 |  |
|  | **N Scot (A)** | **SW Eng (D)** |  |
| Demosponge A | 0.33 ± 0.25 | 1.98 ± 0.91 |  |
| *Mytilus* spp. | 0.44 ± 0.09 | 0.06 ± 0.06 |  |
| *Didemnidae* spp. | 0.18 ± 0.10 | 0.92 ± 0.25 |  |
| Demosponge F | 0.00 ± 0.00 | 0.32 ± 0.10 |  |
| *Verruca stroemia* | 0.68 ± 0.22 | 2.28 ± 0.73 |  |
|  | **W Scot (B)** | **SW Eng (D)** |  |
| *Verruca stroemia* | 0.02 ± 0.01 | 2.28 ± 0.73 |  |
| Demosponge A | 0.00 ± 0.00 | 1.98 ± 0.91 |  |
| *Celleporina caliciformis* | 0.00 ± 0.00 | 0.36 ± 0.08 |  |
| *Didemnidae* spp. | 0.02 ± 0.01 | 0.92 ± 0.25 |  |
| *Balanus crenatus* | 1.17 ± 0.64 | 0.01 ± 0.01 |  |
