# Supplementary material for: Spatial variability in the diversity and structure of faunal assemblages associated with kelp holdfasts (Laminaria hyperborea) in the northeast Atlantic
Source: PLoS One. 2018 Jul 12;13(7):e0200411. doi: 10.1371/journal.pone.0200411 (PMC6042752; doi:10.1371/journal.pone.0200411)
Supplement: S4 Table — Abundance values were fourth-root transformed and standardised by habitable holdfast space prior to analysis. (DOCX) [file pone.0200411.s004.docx]

| **S4 Table. Percentage contributions of individual taxa to observed differences in mobile holdfast assemblages between regions, as determined by SIMPER analysis.** Abundance values were fourth-root transformed and standardised by habitable holdfast space prior to analysis. | | | | | | |
| --- | --- | --- | --- | --- | --- | --- |
| Species | Av. abund | Av. abund | Av. diss | Diss/SD | Contrib% | Cum% |
|  | **N Scot (A)** | **SW Eng (D)** |  | | | |
| *Jassa* spp. | 0.53 | 0.04 | 4.15 | 2.59 | 5.29 | 5.29 |
| *Pisidia longicornis* | 0.01 | 0.32 | 2.60 | 2.03 | 3.31 | 8.60 |
| *Ampithoe* spp. | 0.28 | 0.00 | 2.21 | 1.05 | 2.82 | 11.42 |
| *Branchiomma bombyx* | 0.00 | 0.26 | 2.10 | 1.91 | 2.68 | 14.10 |
| *Lembos websteri* | 0.23 | 0.04 | 1.81 | 1.20 | 2.31 | 16.40 |
|  | **W Scot (B)** | **SW Eng (D)** |  | | | |
| *Jassa* spp. | 0.42 | 0.04 | 3.35 | 1.87 | 4.22 | 4.22 |
| *Pisidia longicornis* | 0.12 | 0.32 | 2.20 | 1.32 | 2.76 | 6.98 |
| *Branchiomma bombyx* | 0.00 | 0.26 | 2.13 | 1.82 | 2.68 | 9.66 |
| *Erichthonius* sp. | 0.26 | 0.06 | 1.92 | 1.45 | 2.41 | 12.07 |
| *Caprella* sp. A | 0.21 | 0.01 | 1.91 | 1.09 | 2.41 | 14.48 |
|  | **Wales (C)** | **SW Eng (D)** |  | | | |
| *Monocorophium sextonae* | 0.42 | 0.04 | 2.85 | 1.69 | 3.73 | 3.73 |
| *Jassa* spp. | **0.41** | 0.04 | 2.85 | 1.53 | 3.73 | 7.45 |
| *Branchiomma bombyx* | 0.00 | 0.26 | 1.91 | 1.90 | 2.50 | 9.96 |
| *Caprella* sp. complex | 0.26 | 0.00 | 1.88 | 0.81 | 2.46 | 12.41 |
| *Lysianassa certatina* | 0.30 | 0.10 | 1.82 | 1.45 | 2.38 | 14.80 |
