# Supplementary material for: Spatial variability in the diversity and structure of faunal assemblages associated with kelp holdfasts (Laminaria hyperborea) in the northeast Atlantic
Source: PLoS One. 2018 Jul 12;13(7):e0200411. doi: 10.1371/journal.pone.0200411 (PMC6042752; doi:10.1371/journal.pone.0200411)
Supplement: S5 Table — (DOCX) [file pone.0200411.s005.docx]

| **S5 Table. Mean abundance values (± SE) for taxa that contributed most to the observed dissimilarities in mobile assemblage structure between regions, as determined by SIMPER (see S4 Table).** | | | |
| --- | --- | --- | --- |
|  | **N Scot (A)** | **SW Eng (D)** |  |
| *Jassa* spp. | 87.3 ± 28.3 | 0.27 ± 0.17 |  |
| *Pisidia longicornis* | 0.05 ± 0.05 | 8.44 ± 2.01 |  |
| *Ampithoe* spp. | 37.2 ± 15.2 | 0.00 ± 0.00 |  |
| *Branchiomma bombyx* | 0.00 ± 0.00 | 3.72 ± 0.63 |  |
| *Lembos websteri* | 5.22 ± 1.20 | 0.22 ± 0.13 |  |
|  | **W Scot (B)** | **SW Eng (D)** |  |
| *Jassa* spp. | 22.1 ± 7.09 | 0.27 ± 0.17 |  |
| *Pisidia longicornis* | 1.27 ± 0.53 | 8.44 ± 2.01 |  |
| *Branchiomma bombyx* | 0.00 ± 0.00 | 3.72 ± 0.63 |  |
| *Erichthonius* sp. | 5.88 ± 1.67 | 0.38 ± 0.18 |  |
| *Caprella* sp. A | 3.16 ± 0.83 | 0.05 ± 0.05 |  |
|  | **Wales (C)** | **SW Eng (D)** |  |
| *Monocorophium sextonae* | 11.2 ± 2.22 | 0.44 ± 0.27 |  |
| *Jassa* spp. | 21.2 ± 10.6 | 0.27 ± 0.17 |  |
| *Branchiomma bombyx* | 0.00 ± 0.00 | 3.72 ± 0.63 |  |
| *Caprella* sp. complex | 15.9 ± 7.26 | 0.00 ± 0.00 |  |
| *Lysianassa certatina* | 5.55 ± 1.70 | 1.50 ± 0.71 |  |
