# Supplementary material for: Spatial variability in the diversity and structure of faunal assemblages associated with kelp holdfasts (Laminaria hyperborea) in the northeast Atlantic
Source: PLoS One. 2018 Jul 12;13(7):e0200411. doi: 10.1371/journal.pone.0200411 (PMC6042752; doi:10.1371/journal.pone.0200411)

**S1 Fig.** **Scatterplots of (a) taxon richness and (b) total biomass/abundance of sessile versus mobile assemblages**. Each data point represents a single holdfast sample (north Scotland = dark blue; west Scotland = light blue; west Wales = pink; southwest England = dark red).


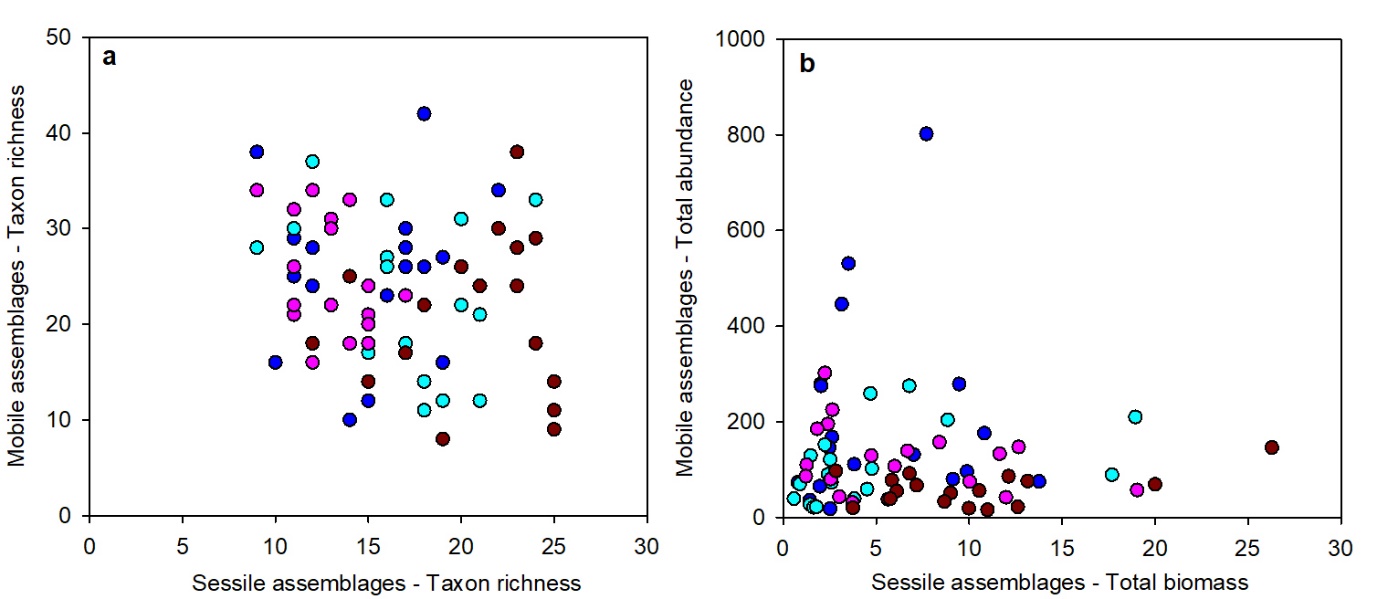

Supplement: S1 Fig — Scatterplots of (a) taxon richness and (b) total biomass/abundance of sessile versus mobile assemblages. Each data point represents a single holdfast sample (north Scotland = dark blue; west Scotland = light blue; west Wales = pink; southwest England = dark red). (DOCX) [file pone.0200411.s006.docx]
